# New Operation for Cleft Palate

**Published:** 1874-07

**Authors:** 


### ARTICLE VIII.

### *New Operation for Cleft in the Hard Palate.*

The following is Sir Wm. Fergusson's operation: —

The first steps of this operation are somewhat similar to the old operation for closing the cleft in the hard palate, namely, paring the edges of the cleft, and making an incision down to the bone, parallel to, and about a quarter of an inch from, the edge of the cleft on either side, the point of the knife being carried back just as far as the junction between the hard and soft palate. Into these incisions a chisel half an inch broad is inserted, and its edge directed against the posterior margin of the hard palate, and made to cut from behind forward, thus partly detaching a slice of bone on each side, with the soft tissues and periosteum attached to their upper and lower surfaces. The result of this is that the sides of the cleft fall easily together, leaving a small aperture through the bone on either side. One, two, or, if the fissure be long, three stitches are passed through the lateral clefts by means of an ordinary aneurism-needle, and thus encircle the detached portions of bone and soft tissue, each suture passing through into the nasal cavity. It should be noted that there is no tension on the flaps, the threads merely keeping the parts steadily in contact. The amount of pain and constitutional disturbance is much less marked in the patients that have been treated in this way than when the old operation of dissecting up the soft parts from the bone has been resorted to.

From the liability of the flaps to twist in slightly, and from the thinness of the edge, Sir William Fergusson is careful to pare the sides somewhat obliquely, in order to present wider raw surface for adhesion. The sutures, which are kept in much longer than in the ordinary operation, cause no harmful irritation. The lateral clefts become filled up by new bone, which is rapidly thrown out, and tends to keep the parts firmly united in the median line.

The first case in which the operation was performed was that of a girl aged eighteen, whose soft palate had been closed two years ago, and whose hard palate had been operated on by the old method three times, but unsuccessfully, except that the gap was somewhat lessened in size. Before the operation by the above plan, on November 22d, 1873, the cleft was half an inch long and a quarter of an inch wide. Two sutures were introduced, and were removed in seven days. She was discharged at the end of the third week, with firm union of the whole palate in the median line, and the lateral clefts closed.—*London Lancet.*

